# A cohort study: The Association Between Autoimmune Disorders and Leptospirosis

**DOI:** 10.1038/s41598-020-60267-0

**Published:** 2020-02-24

**Authors:** Soon-Hian Teh, Ren-In You, Yu-Cih Yang, Chung Y. Hsu, Cheng-Yoong Pang

**Affiliations:** 1 Division of Infectious Disease, Department of Internal Medicine, Hualien Tzu Chi Hospital, Buddhist Tzu Chi Medical Foundation, Hualien, Taiwan; 2 Department of Laboratory Medicine and Biotechnology, College of Medicine, Tzu Chi University, Hualien, Taiwan; 3 Management Office for Health Data, China Medical University Hospital, Taichung, Taiwan; 4 College of Medicine, China Medical University, Taichung, Taiwan; 5 Graduate Institute of Clinical Medical Science, China Medical University, Taichung, Taiwan; 6 Department of Medical Research, Hualien Tzu Chi Hospital, Buddhist Tzu Chi Medical Foundation, Hualien, Taiwan; 7 Cardiovascular and Metabolomics Research Center, Hualien Tzu Chi Hospital, Buddhist Tzu Chi Medical Foundation, Hualien, Taiwan; 8 Institute of Medical Sciences, College of Medicine, Tzu Chi University, Hualien, Taiwan

**Keywords:** Rheumatology, Risk factors

## Abstract

There are limited studies on the association between systemic autoimmune rheumatic diseases (SARDs) and leptospirosis. Therefore, this study aims to identify the effects of leptospirosis on the risks of developing SARDs with a nationwide retrospective cohort study. Patients with leptospirosis who did not have a diagnosis of SARDs before the index date were enrolled from the Taiwan National Health Insurance Research Database between 2000 and 2010, as the leptospirosis cohort. For each patient with leptospirosis, one control without a history of leptospirosis and SARDs was randomly selected (non-leptospirosis cohort). Cox proportional hazards regression models were used to analyze the risk of SARDs according to sex, age, and comorbidities. Among the 23 million people in the cohort, 3,393 patients with leptospirosis (68.91% men, mean age 52.65 years) and 33,930 controls were followed for 18,778 and 232,999 person-years, respectively. The incidence of SARDs was higher in the leptospirosis cohort than in the non-leptospirosis cohort (1.38 vs 0.33 per 1000 person-years), with a hazard ratio (HR) of 4.42 (95% confidence interval [CI] = 2.82–6.92). The risk of developing SARDs was highest for leptospirosis patients aged ≥65 years (HR = 2.81% CI = 1.07–7.36) compared with patients aged ≤39 years. Patients with leptospirosis have a 4.42-fold higher risk of SARDs than that in the general population. Further research is warranted to investigate the mechanism underlying this association.

## Introduction

Leptospirosis is a zoonotic disease caused by pathogenic spirochetes, genus *Leptospira*. Leptospirosis can be found globally, with a higher incidence in tropical and subtropical regions^1^. The pathogen can enter the host through wounds in the skin, the conjunctiva, and mucous membranes. By direct contact with the body fluids from the infected domestic and sylvatic animals, humans may also be infected. Once infected, patients can either be asymptomatic or manifested as an acute febrile illness, with fever, chills, arthralgia, headache, and severe myalgia. Occasionally, it can cause severe disease leading to multiorgan dysfunction and even death^2^. The nonspecific initial presentation and lack of a reliable point-of-care screening test make this disease a diagnostic challenge^3,4^.

Autoimmune diseases are conditions that are triggered by the immune system initiating an attack on self-molecules due to the worsening of immunologic tolerance to auto-reactive immune cells^5^. Both environmental and genetic are significant contributors to the pathogenesis of autoimmune diseases^6^. Autoimmune diseases can either be systemic, organ-specific or localized to specific tissue or region depending on the clinicopathology of each condition. Systemic autoimmune rheumatic diseases (SARDs) are a collective term for more than 80 systemic autoimmune diseases, including systemic lupus erythematosus (SLE), rheumatoid arthritis (RA), and systemic sclerosis (SS), Sjogren’s syndrome, polymyositis, dermatomyositis. Female are inordinately affected, representing nearly 80% of people with autoimmune diseases^7^; for instance, the female to the male ratio of Sjögren’s syndrome or SLE were 9~10:1^8^.

The relationship between infection and autoimmune diseases have been reported. *Borrelia burgdorferi* might induce chronic autoimmune disease called Lyme borreliosis^9^. The infection of Parvovirus B19 has been implicated in the pathogenesis of a variety of autoimmune disorders with the hallmark of inducing the production of autoantibodies in the host^10^. Cytomegalovirus (CMV) infections were correlated with the induction and expansion of a proinflammatory CD4^+^/CD28^−^ T cells in RA patients^11^; and Epstein-Barr virus (EBV) was regarded as an important trigger in the pathogenesis of SLE as evidenced by the co-existence of anti-viral capsid antigen IgG and anti-dsDNA antibodies in the patients^12^. The relationship between leptospirosis and the development of SARDs is unclear; we thus conducted a longitudinal nationwide retrospective cohort study to investigate the occurrence of leptospirosis and the risk of developing SARDs subsequently in the same patients.

## Patients and Methods

### Data source

All data in this study were contained from the database retained by the National Health Research Institute, Taiwan, and were available for public access. Since no identifiable personal information was included in the database, the study was approved by the Research Ethics Committee of China Medical University and Hospital (CMUH-104-REC2-115) and waiver of informed consent was granted as well. The National Health Insurance (NHI) program, which was implemented in 1995, has covered more than 99% of the entire population (23 million residents) of Taiwan. The reliability of the database is secured since false diagnosis reports entail a high penalty. Dataset of the National Health Insurance Research Database (NHIRD) for the year 2000 to 2013 was used, including of registry of beneficiaries, the registry for catastrophic illness, and inpatients care claims released by the Taiwan National Health Research Institutes. In this study, we used the hospitalization claims data of all enrollees in Taiwan, which contained information about sex, birthday, and date of admission and discharge, diagnoses, data of inpatients visits, and diagnosis codes according to the International Classification of Disease, Ninth Revision, Clinical Modification (ICD-9-CM). The medical files in this subset contain all detail medical information of the patients, which include demographics, diagnoses, examinations, and drug prescription.

### Exposure ascertainment

This study was a retrospective cohort study: among 3,393 patients aged 20 years and older who were newly diagnosed with leptospirosis disease (ICD-9-CM code 100.x) as recorded in the inpatients’ registry claim during the year 2000 to 2010. The new diagnosis date of leptospirosis was the index date. No diagnosis of leptospirosis patients was defined non-leptospirosis group (n = 17,308,905). Exclusion criteria included that patients less than 20 years, or missing information of sex or birthday, and diagnosed SARDs date before the index date in both cohort groups. To bolster comparability between the case of leptospirosis and non-leptospirosis patients, we randomly selected 1:10 ratios of leptospirosis and non-leptospirosis from each stratum of a combination of sex, age (per 5 years), and index years.

### Study outcome

The primary outcome, newly diagnosis of SARDs, is one of the 31 categories of serious illnesses or injuries that subject the patients to Catastrophic Illness Certificate. SARDs is consisted of SLE (ICD-9-CM: 710.0), SS (ICD-9-CM: 710.1), RA (ICD-9-CM: 714.0), polyarticular juvenile RA (ICD-9-CM: 714.30~714.33), polymyositis (ICD-9-CM: 710.4), dermatomyositis (ICD-9-CM: 710.3), vasculitis (included polyarteritis nodosa, ICD-9-CM: 446.0), hypersensitivity angiitis (ICD-9-CM: 446.2), Wegener’s granulomatosis (ICD-9-CM: 446.4), giant cell arteritis (ICD-9-CM: 446.5), thromboangiitis obliterans (ICD-9-CM: 443.1), Takayasu’s disease (ICD-9-CM: 446.7), acute febrile mucocutaneous lymph node syndrome (ICD-9-CM: 446.1), Behcet’s syndrome (ICD-9-CM: 136.1), pemphigus (ICD-9-CM: 694.4), sicca syndrome (ICD-9-CM: 710.2), Crohn’s disease (ICD-9-CM: 555), and ulcerative colitis (ICD-9-CM: 556.0~556.6, 556.8~556.9). Each participant was followed up from index date to the date of incident SARDs, or withdrawal from NHI, or until December 31, 2013, whichever occurred first. Patients with inpatients’ claim reporting a history of diabetes mellitus (ICD-9-CM: 250.x), hypertension (ICD-9-CM: 401–405), coronary artery disease (ICD-9-CM: 410–414), and cerebrovascular disease (ICD-9-CM: 430–438) identified before the index date, were considered as potential confounders in this study.

### Statistical analysis

The descriptive table was used to examine the baseline characteristics of the study cohort groups. We used the chi-square test for categorical variables and the t-test for continuous variables. We evaluated the Hazard Ratios (HRs) and 95% confidence interval (95% CI) for each variable using the Cox proportional hazards model. We estimated the cumulative risk of SARDs for both cohort by using the Kaplan-Meier method, and the log-rank test was used to assess the significance of the cumulative risk curves. All statistical analyses were performed with SAS statistical analysis software, version 9.4 (SAS Institute Inc, Cary, North Carolina), and the significance level was set at 0.05.

## Result

### Characteristics of the participants

A total of 3,393 patients with leptospirosis and 33,930 matched patients without leptospirosis were included in the study. The mean (±SD) ages of the leptospirosis cohort and non-leptospirosis cohort were 52.65 (±16.52) years old and 52.59 (±16.57) years old, respectively. The proportion of male and female were 68.91% and 31.09%, respectively. The proportion of diabetes, hypertension, coronary artery disease and cerebrovascular disease in leptospirosis cohort were higher than non-leptospirosis cohort. Table 1 summarized the demographic characteristics and baseline comorbidities of the subjects. The mean (median) of the follow-up periods was 5.53 (5.63) years and 6.87 (7.00) years for leptospirosis cohort and compared cohort group, respectively.

**Table 1:** Demographic characteristics of the patients with and without leptospirosis in Taiwan.

Variable	Leptospirosis				P-value
	No		Yes		
	n = 33930		n = 3393		
	n	%	n	%	
Sex					0.99*
Female	10550	31.09	1055	31.09	
Male	23380	68.91	2338	68.91	
Age at baseline, years					0.99*
20–39	8620	25.41	862	25.41	
40–64	16510	48.66	1651	48.66	
Older than 65	8800	25.94	880	25.94	
Mean (±SD)	52.59 (±16.57)		52.65 (±16.52)		0.8389^‡^
**Baseline comorbidities**					
Diabetes	1833	5.40	380	11.20	<0.0001*
Hypertension	3339	9.84	554	16.33	<0.0001*
Coronary artery disease	1533	4.52	233	6.87	<0.0001*
Cerebrovascular disease	1507	4.44	240	7.07	<0.0001*

^‡^t test; *chi-square test.

### Associations in subgroups

**Figure 1:**
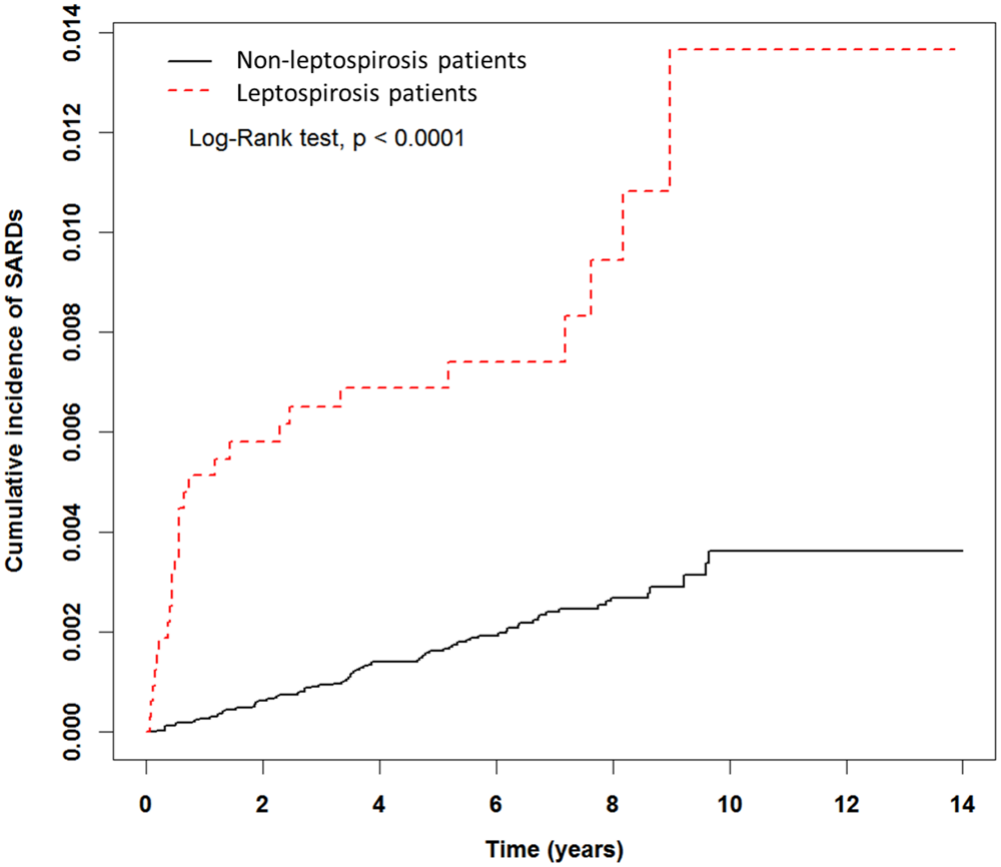
Cumulative incidence of SARDs for leptospirosis cohort and non-leptospirosis cohort.

The leptospirosis patients had a higher cumulative incidence of developing SARDs when compared to the comparison cohort during the follow-up period (Log-rank test, p < 0.0001, Fig. 1). Table 2 shows the uni- and multivariate Cox’s proportional hazard model. In univariate analysis, there was a 4.16-fold higher risk of SARDs in the study cohort compared with the comparison cohort (crude HR = 4.16, 95% CI = 2.67–6.50). After adjusted for age, gender, and comorbidities (included diabetes, hypertension, coronary artery disease, and cerebrovascular disease), leptospirosis patients had a higher risk of SARDs than the non-leptospirosis patients (adjusted HR = 4.42, 95% CI = 2.82–6.92).

**Table 2:** Cox model with hazard ratios and 95% confidence intervals of SARDs associated with and without leptospirosis.

Variable	SARDs	Crude^*^			Adjusted^†^		
	no. (n = 103)	HR	(95% CI)	p-value	HR	(95% CI)	p-value
**Leptospirosis**							
No	77	1.00	reference		1.00	reference	
Yes	26	4.16	(2.67–6.50)	<0.0001	4.42	(2.82–6.92)	<0.0001
**Sex**							
Female	61	1.00	reference		1.00	reference	
Male	42	0.31	(0.21–0.46)	<0.0001	0.33	(0.22–0.48)	<0.0001
**Age at baseline, years**							
20–39 years	13	1.00	reference		1.00	reference	
40–64 years	59	2.38	(1.31–4.34)	0.0046	2.30	(1.26–4.20)	0.0068
Older than 65 years	31	2.67	(1.39–5.10)	0.0030	2.69	(1.38–5.25)	0.0036
**Baseline comorbidities**							
Diabetes	3	0.60	(0.19–1.89)	0.3828	0.45	(0.14–1.49)	0.1914
Hypertension	8	0.89	(0.43–1.83)	0.7465	0.71	(0.31–1.65)	0.4295
Coronary artery disease	4	1.00	(0.37–2.71)	0.9956	1.01	(0.34–2.98)	0.9812
Cerebrovascular disease	4	1.05	(0.39–2.84)	0.9287	1.04	(0.35–3.06)	0.9464

^*^Represents relative hazard ratio (HR); ^†^represents adjusted HR: mutually adjusted for leptospirosis, age, gender, and baseline comorbidities (diabetes, hypertension, coronary artery disease, and cerebrovascular disease) in Cox proportional hazard regression. Abbreviations: SARDs, systemic autoimmune rheumatic diseases; CI, confidence interval.

### Incidence rate

We determined the incidence of SARDs in leptospirosis and non-leptospirosis cohorts, and the result indicated that patients with leptospirosis had a higher significant risk of SARDs (Table 3). Incidence rates (per 1,000 person-years) of SARDs were 1.38 and 0.33 for leptospirosis cohort and non-leptospirosis cohort, respectively. The incidence of SARDs with leptospirosis cohort were 2.55 and 0.85 (per 1,000 person-years) for female and male, respectively, which was higher than non-leptospirosis cohort. Besides, female showed 4.37-fold (95% CI: 2.43–7.87), and male showed 4.63-fold (95% CI: 2.31–9.26) higher risk of developing SARDs than the compared cohort. Leptospirosis had a higher risk of SLE (adjusted HR: 10.48, 95% CI: 3.47–31.67) and SS (adjusted HR: 10.15, 95% CI: 1.62–63.71) than non-leptospirosis cohort (Supplementary Table 1).

**Table 3:** Incidence and Cox proportional hazard regression with hazard ratios and 95% confidence intervals of SARDs associated with and without leptospirosis by gender and age group.

Variable	Non-leptospirosis			Leptospirosis			Crude HR (95% CI)	Adjusted HR^‡^ (95% CI)
	Event	Person-years	IR^†^	Event	Person-years	IR^†^		
Total	77	232999	0.33	26	18778	1.38	4.16 (2.67–6.50)***	4.42 (2.82–6.92)***
**Gender**								
Female	46	72241	0.64	15	5872	2.55	3.93 (2.19–7.05)***	4.37 (2.43–7.87)***
Male	31	160758	0.19	11	12905	0.85	4.49 (2.25–8.94)***	4.63 (2.31–9.26)***
**Age group, year**								
20–39 years	6	61587	0.10	7	5256	1.33	12.88 (4.33–38.33)***	13.33 (4.48–39.68)***
40–64 years	45	116966	0.38	14	9632	1.45	3.80 (2.08–6.93)***	3.82 (2.08–7.03)***
Older than 65 years	26	54446	0.48	5	3890	1.29	2.68 (1.03–6.98)*	2.81 (1.07–7.36)*

^†^Incidence rates (IR) per 1,000 person-years; ^‡^represents adjusted hazard ratio (HR): mutually adjusted for leptospirosis, age, gender and baseline comorbidities (diabetes, hypertension, coronary artery disease, and cerebrovascular disease) in Cox proportional hazard regression. *<0.05; **<0.01; ***p < 0.001. The reference group was a non-leptospirosis group. Abbreviations: SARDs, systemic autoimmune rheumatic diseases; CI, confidence interval.

## Discussion

The etiology of the majority of autoimmune diseases is not well-defined. Some factors, which include genetics, environmental toxins, and the microbiome, can influence or induce autoimmunity^13–15^. The inappropriate immune activation which targets to self-antigens causes sustainable pathologies that make an individual subjected to a various grade of autoimmune diseases. Some pathogens have been reported to be associated with the pathogenesis of autoimmune disorders^16,17^. These infectious agents trigger autoimmune diseases via various mechanisms. Many bacterial infections have been related to the development of autoimmune-like response and resulting as an essential clinical problem^18^.

The proposed mechanisms include molecular mimicry, which antibodies against bacterial antigens that are similar to the host^19^. For example, autoimmune diseases such as Crohn’s disease, ulcerative colitis, and sarcoidosis are commonly found in *Mycobacterium tuberculosis* patients^20^. *Streptococcus pyogenes* infection can lead to rheumatic heart disease, while *Chlamydia* and *Campylobacter* bacteria cause reactive arthritis^21^. These associations between various bacterial infections and SARDs consist of the observation herein that leptospiral infection is a crucial factor for the risk of autoimmune disease. It has been proposed that antibiotics that are customarily prescribed for treating infectious pathogens can offer effects upon curing/alleviating autoimmune diseases^22,23^.

To date, there have been few studies evaluating the exposure of spirochete infection in newly diagnosed autoimmune disease. A high frequency of *Borrelia burgdorfeii* infections complicating autoimmune symptom, especially arthritis, has been reported previously^24^. *Treponema denticola* causes periodontitis and induces RA^25^. The other type of spiral bacteria-*Campylobacter jejuni* has also been reported to be associated with an autoimmune post-infectious polyradiculoneuropathy called Guillain Barre Syndrome^26^. Our study showed that SARDs occurred more frequently among patients with leptospirosis. Using a nationwide retrospective cohort database, our study is the first to reveal that patients with leptospirosis exhibited a 4.42-fold greater risk of developing subsequent SARDs than that of the general population (Table 2). Our findings are etiologic importance, which may offer useful predictive information for future clinical management.

The prevalence of autoimmune diseases in the female is higher than male^27,28^. In this report, we found that the HR of SARDs associated with leptospirosis is similar in female and male (Table 3, F/M: 4.37/4.63). The incidence ratio (IR) of SARDs in both non-leptospirosis (Table 3, F/M:0.64/0.19) and leptospirosis (Table 3, F/M: 2.55/0.85) are higher in the female. Some reports suggested that sex hormones might mediate the immune response by alternating T cell development and function^29^. Sex-based changes due to environmental factors or exposure to sex hormones could modify the induction of various autoimmune diseases^27,28,30^. Thus, it is very important to elucidate whether sex hormones are among the risk factors in leptospirosis patients that subsequently developing SARDs.

We further demonstrated that age was an independent predictor for newly diagnosed SARDs in patients with leptospirosis (Table 3). Previous studies demonstrated a more severe symptom of leptospirosis in adult as compared with infected children, and male predominance of severe leptospirosis in children and adolescents was also noted^31^. It is noteworthy that both analyses identified age as an independent variable associated with an increased risk of leptospirosis. We further identified a significantly high risk of young age with leptospirosis associated SARDs (Table 3. Crude HRs of age 20/40/65:12.88/3.8/2.68). In leptospiral infection, it was reported that patients with mild leptospirosis had a background level of pre-existing IgG, while patients with severe leptospirosis demonstrated a gradual increase of IgM antibody profile typical of first exposure^32^. Pre-existing IgG against *leptospira* is suspected to be protective against severe clinical manifestations.

In general, leptospiral infection is associated with increased susceptibility to SARDs. However, no sufficient data exists for the effect of age on the risk of developing SARDs; our study demonstrated that the presence of the leptospiral infection in elderly with SARDs. The mechanism may be due to abnormalities of the host immune response, particularly immune senescence, resulted in many inflammatory responses, increasing susceptibility to infections, and enhancing the production of autoantibodies^33^. Another possible explanation is the expansion of many protective regulatory mechanisms in the elderly (e.g., higher production of peripheral T-regulatory cells), resulting in higher autoimmunity but yet a lower prevalence of autoimmune diseases^34^. The decrease of thymic T-regulatory cells has been reported in association with the loss of thymic capacity to generate new T cells in the elderly^35^, which could be a hint that people with older age tend to have an inadequacy in response to pathogens. These premises need to be explored in the future by recruiting more younger patients for additional analysis.

### Limitation

The present study is not without its limitations: firstly, we have performed analysis by disease subtypes-SLE, RA, vasculitis, and Sicca syndrome, in our non-leptospirosis and leptospirosis cohorts (Supplementary Table 1), however, the sample size is too small to reach an apparent conclusion. We found that leptospirosis had a higher risk of SLE (adjusted HR: 10.48, 95% CI: 3.47–31.67), and SS (adjusted HR: 10.15, 95% CI: 1.62–63.71) than non-leptospirosis cohort. Secondly, no data were available on medical history, lifestyle, and vaccination status of the enrollees, raising concerns about the involvement of other residual confounding factors. Thirdly, leptospirosis is diagnosed on clinical grounds and confirmed by microscopic agglutination test (MAT) or polymerase chain reaction (PCR) practically. However, the retrieval data from the National Health Insurance Database is based on clinical diagnosis (ICD-9 code) judged by the physicians; thus, it is not a confirmed diagnosis. The correlation between leptospirosis and the subsequent SARDs has to be validated in the future.

## Supplementary information

Supplementary Table 1.
