# Supplementary material for: A cohort study: The Association Between Autoimmune Disorders and Leptospirosis
Source: Sci Rep. 2020 Feb 24;10:3276. doi: 10.1038/s41598-020-60267-0 (PMC7039877; doi:10.1038/s41598-020-60267-0)
Supplement: Supplementary file 1 — Supplementary Table 1. [file 41598_2020_60267_MOESM1_ESM.docx]

**A cohort study: The Association Between Autoimmune Disorders and Leptospirosis**

Soon-Hian Teh*^a^, Ren-In You*^b^, Yu-Cih Yang^c,d^, Chung Y. Hsu^e^, Cheng-Yoong Pang^#f,g,h^

^a^ Division of Infectious Disease, Department of Internal Medicine, Hualien Tzu Chi Hospital, Buddhist Tzu Chi Medical Foundation, Hualien, Taiwan.

^b^ Department of Laboratory Medicine and Biotechnology, College of Medicine, Tzu Chi University, Hualien, Taiwan.

^c^ Management Office for Health Data, China Medical University Hospital, Taichung, Taiwan.

^d^ College of Medicine, China Medical University, Taichung, Taiwan.

^e^ Graduate Institute of Clinical Medical Science, China Medical University, Taichung, Taiwan.

^f^ Department of Medical Research, Hualien Tzu Chi Hospital, Buddhist Tzu Chi Medical Foundation, Hualien, Taiwan.

^g^ Cardiovascular and Metabolomics Research Center, Hualien Tzu Chi Hospital, Buddhist Tzu Chi Medical Foundation, Hualien, Taiwan.

^h^ Institute of Medical Sciences, College of Medicine, Tzu Chi University, Hualien, Taiwan.

* These authors contributed equally.

^#^ Correspondence:

Dr. Cheng-Yoong Pang, Department of Medical Research, Hualien Tzu Chi Hospital, Buddhist Tzu Chi Medical Foundation, Hualien, Taiwan. Address: Room 613, Xie-Li Building, No 707, Sec 3, Chung-Yang Road, 97002, Hualien, Taiwan. Tel: +886-38561825 Ext. 15613. Fax: +886-38573710. Email: cypang@mail.tcu.edu.tw.

Supplementary Table 1. Incidence of type of SARDs among patients with and without leptospirosis.

| SARDs | Non-leptospirosis | | |  | | Leptospirosis | | | |  | Crude HR  (95%CI) | | Adjusted HR‡  (95%CI) |
| --- | --- | --- | --- | --- | --- | --- | --- | --- | --- | --- | --- | --- | --- |
|  | Event | Person-years | IR^†^ | |  | | Event | Person-years | IR^†^ |  |  |  |  |
| Systemic lupus erythematosus | 7 | 232999.6 | 0.03 | |  | | 6 | 18777.6 | 0.32 |  |  | 10.74 (3.59-32.08)*** | 10.48 (3.47-31.67)*** |
| Systemic sclerosis | 3 | 232999.0 | 0.01 | |  | | 2 | 18778.2 | 0.11 |  |  | 9.24 (1.51-56.48)* | 10.15 (1.62-63.71)* |
| Rheumatoid arthritis | 42 | 233000.4 | 0.18 | |  | | 8 | 18777.6 | 0.43 |  |  | 1.78 (0.81-3.90) | 1.64 (0.67-3.97) |
| Polymyositis | 2 | 232999.0 | 0.01 | |  | | 0 | 18777.6 | 0.00 |  |  | - | - |
| Dermatomyositis | 0 | 232999.0 | 0.00 | |  | | 1 | 18777.6 | 0.05 |  |  | - | - |
| Vasculitis | 1 | 232999.3 | 0.00 | |  | | 7 | 18778.0 | 0.37 |  |  | 2.05 (0.23-18.24) | 10.04 (0.47-216.31) |
| Pemphigus | 1 | 232999.0 | 0.00 | |  | | 1 | 18777.6 | 0.05 |  |  | - | - |
| Sicca syndrome | 26 | 232999.0 | 0.11 | |  | | 4 | 18778.1 | 0.21 |  |  | 1.92 (0.67-5.51) | 1.98 (0.69-5.70) |
| Crohn's disease | 0 | 232999.0 | 0.00 | |  | | 0 | 18777.6 | 0.00 |  |  | - | - |
| Ulcerative colitis | 2 | 232999.0 | 0.01 | |  | | 0 | 18777.6 | 0.00 |  |  | - | - |

^†^ incidence rates per 1,000 person-years; ^‡^ represents adjusted hazard ratio: mutually adjusted for leptospirosis, age, gender and baseline comorbidities (diabetes, hypertension, coronary artery disease, and cerebrovascular disease) in Cox proportional hazard regression. *:<0.05; **:<0.01; *** p<0.001. The reference group was a non-leptospirosis group. Abbreviations: SARDs, systemic autoimmune rheumatic diseases; HR, hazard ratio; CI, confidence interval.
